# Ubiquitin phosphorylation in Parkinson’s disease: Implications for pathogenesis and treatment

**DOI:** 10.1186/s40035-015-0049-6

**Published:** 2016-01-06

**Authors:** Lih-Shen Chin, Lian Li

**Affiliations:** Department of Pharmacology and Center for Neurodegenerative Disease, Emory University School of Medicine, Atlanta, GA 30322 USA

**Keywords:** Mitophagy, Parkinson’s disease, PINK1, Parkin, Ubiquitin-protein ligase, Ubiquitin phosphorylation, Mitochondrial quality control, Mitophagy

## Abstract

Parkinson’s disease (PD) is the most common neurodegenerative movement disorder, characterized primarily by the loss of dopaminergic neurons in substantia nigra. The pathogenic mechanisms of PD remain unclear, and no effective therapy currently exists to stop neurodegeneration in this debilitating disease. The identification of mutations in mitochondrial serine/threonine kinase PINK1 or E3 ubiquitin-protein ligase parkin as the cause of autosomal recessive PD opens up new avenues for uncovering neuroprotective pathways and PD pathogenic mechanisms. Recent studies reveal that PINK1 translocates to the outer mitochondrial membrane in response to mitochondrial depolarization and phosphorylates ubiquitin at the residue Ser65. The phosphorylated ubiquitin serves as a signal for activating parkin and recruiting autophagy receptors to promote clearance of damaged mitochondria via mitophagy. Emerging evidence has begun to indicate a link between impaired ubiquitin phosphorylation-dependent mitophagy and PD pathogenesis and supports the potential of Ser65-phosphorylated ubiquitin as a biomarker for PD. The new mechanistic insights and phenotypic screens have identified multiple potential therapeutic targets for PD drug discovery. This review highlights recent advances in understanding ubiquitin phosphorylation in mitochondrial quality control and PD pathogenesis and discusses how these findings can be translated into novel approaches for PD diagnostic and therapeutic development.

## Background

Parkinson’s disease (PD) is the most common neurodegenerative movement disorder with a prevalence of about 1 % at the age of 65 and of 4 %–5 % by the age of 85 [1, 2]. The disease occurs either in relatively rare, familial forms or in common, sporadic forms [3]. The different forms of PD share similar motor symptoms of rigidity, bradykinesia, postural instability, and resting tremor, which appear when there is a loss of 50 %–60 % of dopaminergic neurons in the substantia nigra pars compacta. Increasing evidence indicates that neurodegeneration is more widespread and occurs in multiple regions in the brain [4, 5]. The etiology of PD, particularly sporadic PD cases, is unknown, and there is no reliable biomarker for PD diagnosis. Current medications for PD only provide temporary relief of motor symptoms with no disease-modifying activity to delay or stop disease progression [3, 6]. Thus, there is clearly a need to develop new diagnostic approaches and more effective therapeutics for PD.

Although familial forms of PD account for less than 10 % of PD cases, the discovery of genes responsible for familial PD cases has provided insights into pathogenic mechanisms leading to neurodegeneration in PD. For example, the identification of loss-of-function mutations in mitochondrial serine/threonine kinase PINK1 as a cause of familial PD [7–9] provides genetic evidence for an involvement of mitochondrial dysfunction in PD pathogenesis. The finding of loss-of-function mutations in E3 ubiquitin-protein ligase parkin as a cause of familial PD [9–11] indicates a role of ubiquitination dysregulation in PD pathogenesis. Ubiquitination is a dynamic post-translational modification in which ubiquitin, a 76-amino-acid polypeptide, is conjugated to a lysine residue in substrate proteins through coordinated sequential actions of E1 ubiquitin-activating enzyme, E2 ubiquitin-conjugating enzyme, and E3 ubiquitin-protein ligase [12]. Proteins can be either monoubiquitinated or polyubiquitinated via successive conjugation of additional ubiquitin molecules to one of the seven internal lysine residues in the preceding ubiquitin. The different types of ubiquitination play distinct signaling roles in regulation of diverse cellular processes by modulating protein activity, localization, trafficking, or degradation [13, 14]. Ubiquitin-dependent signalling is also modulated by deubiquitinating enzymes, which catalyze the removal of ubiquitin from proteins [15]. Recent phosphoproteomic studies revealed that ubiquitin itself can be phosphorylated [16–18], adding a new layer of control over the ubiquitin signalling system. Interestingly, PINK1 was identified as a ubiquitin kinase for phosphorylation of ubiquitin, and the phosphorylated ubiquitin was shown to play novel signaling roles in activating parkin and recruiting autophagy receptors to promote mitophagy. In this review, we will summarize recent findings on the roles of ubiquitin phosphorylation in mitochondrial quality control and PD pathogenesis. We will also discuss the potential of ubiquitin phosphorylation as a PD biomarker and the strategies to target ubiquitin phosphorylation-dependent mitophagy for PD therapeutic intervention.

### PINK1 phosphorylates ubiquitin at Ser65 in response to mitochondrial depolarization

Mitochondria are double membrane-bound organelles with four distinct submitochondrial compartments: the outer mitochondrial membrane (OMM), the inner mitochondrial membrane (IMM), the intermembrane space (IMS), and the matrix. The compartmentalization is crucial to mitochondria-mediated processes, including energy production, metabolism, redox control, calcium homeostasis, and programmed cell death [19, 20]. Mitochondrial dysfunction is implicated as a key factor in PD pathogenesis [21–23]. Human genetic studies revealed that homozygous mutations in mitochondrial kinase PINK1 cause autosomal recessive, early-onset PD [7–9, 24], whereas heterozygous mutations in PINK1 increase the risk for developing late-onset PD [25–27], highlighting the importance of knowing the sites and mechanisms of PINK1 action.

**Fig. 1:**
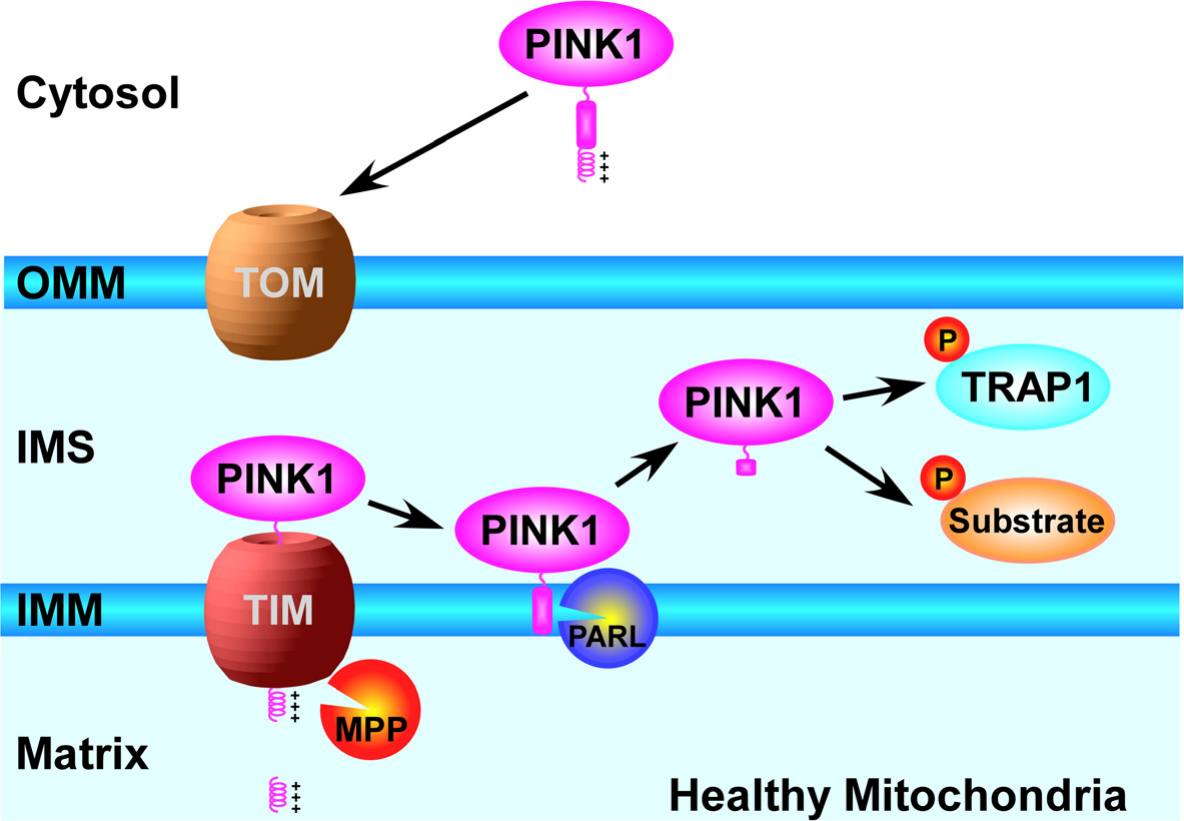
PINK1-mediated intramitochondrial signaling in healthy mitochondria. PINK1 is imported into healthy mitochondria through the TOM and TIM complexes and is then cleaved sequentially by mitochondrial processing peptidase (MPP) and PARL to generate a processed form of PINK1 that resides in the intermembrane space. There, PINK1 can phosphorylate mitochondrial chaperone TRAP1 and perhaps also other substrates to regulate the activities of polarized mitochondria, such as respiration and redox control

PINK1 is an ubiquitously expressed, 581-amino-acid protein Ser/Thr kinase with an N-terminal mitochondrial targeting sequence [28]. Under normal physiological conditions, PINK1 is imported into healthy mitochondria through the translocase of outer membrane (TOM) and translocase of inner membrane (TIM) complexes [29], where the 64-kDa full-length PINK1 can undergo sequential proteolytic cleavages by the matrix-localized mitochondrial processing peptidase (MPP) and the IMM-localized protease PARL to generate a 52-kDa processed form of PINK1 [30–33]. According to one model, the 52-kDa processed form of PINK1 is retrotranslocated to the cytosol for rapid degradation by the proteasome through the N-end rule pathway [34] and consequently, PINK1 protein levels are virtually undetectable under normal conditions, thus arguing against a function of PINK1 in healthy mitochondria [30, 34, 35]. In contrast, other studies reported significant levels of PINK1 protein under normal conditions, but localized PINK1 to either the OMM with its kinase domain facing the cytoplasm [36–38] or to the IMM/IMS with its kinase domain facing the IMS [39–43]. Recently, super-resolution imaging analyses using three-dimensional structured illumination microscopy (3D-SIM) [44] or a combination of tracking and localization microscopy (TALM) and fluorescence photoactivation localization microscopy (F-PALM) [45] clearly showed that, under normal conditions, PINK1 is not present on the OMM of healthy mitochondria, but rather PINK1 resides in the IMM/IMS where it is mainly localized to the cristae membrane and intracristae space. Furthermore, PINK1 was found to colocalize with the mitochondrial chaperone TRAP1, a previously identified PINK1 substrate [40, 46], in these submitochondrial compartments [44]. Together, these results support a model (Fig. 1) that PINK1 plays an intramitochondrial signaling role by phosphorylating TRAP1 [40] and potentially also other IMM/IMS-localized proteins, such as the complex I subunit NdufA10 [47] and the mitochondrial serine protease HtrA2 [48], to regulate activities of polarized mitochondria.

Super-resolution imaging analyses showed that, in response to mitochondrial depolarization, PINK1 changes its submitochondrial localization from the IMM/IMS to the OMM of depolarized mitochondria [44, 45], whereas TRAP remains in the IMM/IMS [44]. The PINK1 localization on the OMM of depolarized mitochondria is likely due to the blockade of PINK1 mitochondrial import through the IMM by the loss of mitochondrial membrane potential [29]. As expected, the mitochondrial import blockade prevents the cleavage of PINK1 by PARL, leading to accumulation of full-length PINK1 on damage mitochondria [29, 30, 33]. Recently, accumulation of misfolded proteins in the mitochondrial matrix was reported to cause PINK1 localization on the OMM without mitochondrial depolarization [49], which might be explained by the possibility that misfolded proteins may somehow inhibit PINK1 mitochondrial import through the TIM complex. Increasing evidence supports that mitochondrial dysfunction-triggered PINK1 localization on the OMM serves as a damage-sensing, quality-control mechanism to mark damaged mitochondria for clearance by mitophagy [29, 35, 44, 49, 50].

Mitochondrial depolarization not only causes PINK1 localization on the OMM of damaged mitochondria but also induces PINK1 dimerization and autophosphorylation at its Ser228 and Ser402 residues [51, 52], which could be an activation mechanism for enhancing PINK1 kinase activity [53]. Intriguingly, PINK1 was recently found to phosphorylate the residue Ser65 of either ubiquitin [54–57] or ubiquitin chains conjugated to mitochondrial proteins in response to mitochondrial depolarization [57, 58], indicating a function of PINK1 as a ubiquitin kinase. This finding is very exciting because previous phosphoproteomic analyses revealed that ubiquitin can be phosphorylated at multiple sites, including Ser65 [16, 17, 59], but the identity of the kinases for phosphorylating ubiquitin was unknown. Quantitative proteomic analysis showed that, under normal conditions, the Ser65-phosphorylated form of ubiquitin (phospho-Ser65-ubiquitin) is essentially undetectable on healthy mitochondria, but upon mitochondrial depolarization, the level of phospho-Ser65-ubiquitin increases to ~20 % of the total ubiquitin level on damaged mitochondria [57], indicating ubiquitin phosphorylation at Ser65 is a stress-responsive signal that can be induced by mitochondrial dysfunction. Interestingly, a study in yeast [18] demonstrated that ubiquitin phosphorylation at Ser65 can also be induced by oxidative stress, although another kinase must be involved because no PINK1 orthologue exists in yeast. Emerging data indicate that ubiquitin phosphorylation at Ser65 causes significant changes in the structures of ubiquitin and ubiquitin chains and affects ubiquitination and deubiquitination cascades catalyzed by a number of E2 ubiquitin-conjugating enzymes, E3 ligases, and deubiquitinating enzymes [18, 60]. Thus, ubiquitin phosphorylation can have a profound impact on the ubiquitin signalling system. Below, we will focus on the role of PINK1-mediated ubiquitin phosphorylation in activating parkin to promote mitophagy.

### Ser65-phosphorylated ubiquitin activates parkin and recruits autophagy receptors on damaged mitochondria to promote mitophagy

There has been intense interest in understanding parkin-regulated neuroprotective processes because loss-of-function mutations in parkin are a major cause of familial PD [9–11] and oxidative/nitrosative stress-induced damage to parkin is associated with sporadic PD [61–63]. Parkin is a 465-amino-acid, cytosolic E3 ubiquitin-protein ligase with an N-terminal ubiquitin-like (Ubl) domain and four zinc-binding domains, RING0, RING1, IBR (in-between RING), and RING2 [64]. Parkin is expressed in many tissues and cell types, where it localizes in the cytosol under normal physiological conditions [10, 11]. *Drosophila* genetic studies provided first evidence that parkin functions downstream of PINK1 in a common pathway involved in the maintenances of mitochondrial homeostasis [65, 66]. Subsequent studies in mammalian cells showed that PINK1 is required for recruiting parkin from the cytosol to depolarized mitochondria to promote mitophagy [35, 37, 50, 67, 68]. Biochemical analyses revealed that parkin is a PINK1 substrate and identified the residue Ser65 within the Ubl domain of Parkin as the phosphorylation site by PINK1 [69–71].

**Fig. 2:**
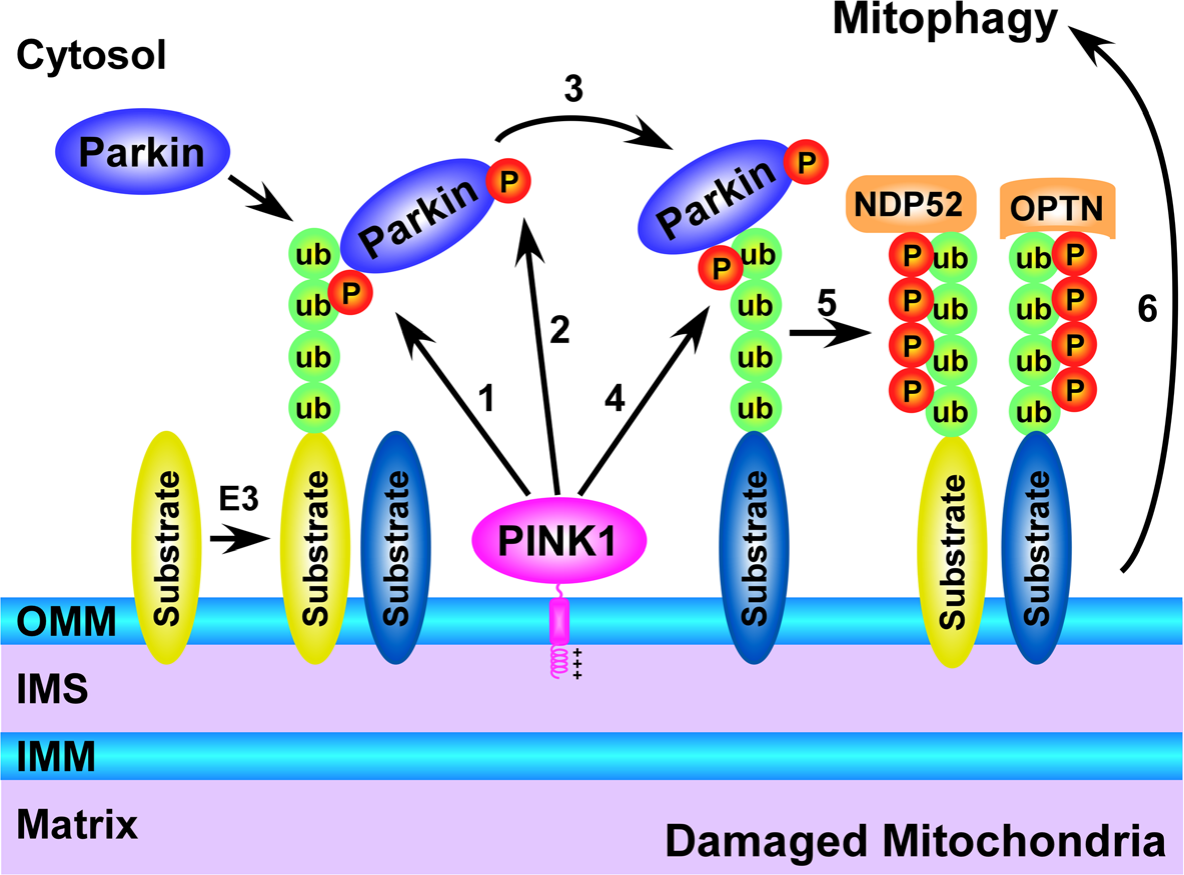
PINK1-mediated phosphorylation of ubiquitin and parkin on damaged mitochondria in facilitation of mitophagy. Mitochondrial damage causes PINK1 localization and activation on the OMM, leading to Ser65-phosphorylation of pre-existing ubiquitin chains conjugated to OMM proteins (*1*). The phosphorylated ubiquitin recruits parkin, enables Ser65-phosphorylation of parkin by PINK1, and activates parkin (*2*). The activated parkin ubiquitinates additional OMM proteins (*3*) and thus provides more substrates for phosphorylation by PINK1 (*4*), leading to further recruitment and activation of parkin (*2*). This positive feed-forward cycle results in a rapid increase in the local concentration of Ser65-phosphorylated ubiquitin *(5)*, which serves as a signal for recruiting autophagy receptors, such as OPTN and NDP52, to promote mitophagy *(6)*

Convergent data from recent studies support a model that PINK1-mediated ubiquitin phosphorylation and parkin phosphorylation work in concert to activate parkin and recruit autophagy receptors to promote mitophagy (Fig. 2). Mitochondrial dysfunction triggers PINK1 localization and activation on the OMM of damaged mitochondria [29, 35, 44, 49, 50], resulting in Ser65-phosphorylation of ubiquitin chains that are already conjugated to OMM proteins by a yet unidentified E3 ligase(s) [51–53]. A function of Ser65-phosphorylated ubiquitin is to serve as a parkin receptor for binding and recruiting parkin from the cytosol to the OMM of damaged mitochondria [58]. Ser65-phosphorylated ubiquitin also functions as an allosteric activator of parkin E3 ligase activity [72–75]. Structural analyses showed that parkin normally exists in an inactive or autoinhibited conformation [64, 76] and that binding of Ser65-phosphorylated ubiquitin to parkin causes a substantial conformational change in parkin, which together with Ser65-phosphorylation parkin Ubl domain, converts parkin from the inactive conformation to an active conformation [72–75]. Once activated, parkin is able to ubiquitinate many OMM proteins [77, 78], which in turn provide additional substrates for phosphorylation by PINK1, leading to further recruitment and activation of parkin, thereby acting as a positive-feedback amplification mechanism to dramatically increase the local concentration of Ser65-phosphorylated ubiquitin on damaged mitochondria [57, 79]. In addition, Ser65-phosphorylated ubiquitin chains are resistant to deubiquitination by many deubiquitinating enzymes, including USP15 and USP30 [60], thereby further contributing to the accumulation of Ser65-phosphorylated ubiquitin on damaged mitochondria. Importantly, a recent study showed that the Ser65-phosphorylated ubiquitin on damaged mitochondria serves as a signal for recruiting autophagy receptors, such as optineurin (OPTN) and NDP52, which then recruit the components of the autophagy machinery to promote autophagic clearance of damaged mitochondria [80].

### Dysregulation of ubiquitin phosphorylation in PD pathogenesis

Human genetic studies have identified numerous PD-causing, homozygous mutations in PINK1 and parkin, which are distributed throughout all domains of these two proteins [7–11]. Recent evidence from studying PD-linked PINK1 and parkin mutations indicates that impairment in ubiquitin phosphorylation-dependent mitochondrial quality control is critically involved in PD pathogenesis. A number of PD-linked mutations found in the PINK1 kinase domain, such as PINK1 G309D, L347P, C388R, and G409V mutations, have been shown to abolish the kinase activity of PINK1 for phosphorylating its substrates and the ability of PINK1 to promote parkin recruitment [40, 69, 81, 82], indicating that mutation-induced loss of PINK1 catalytic activity is a mechanism leading to impaired mitophagy and neurodegeneration. In addition, PD-linked C92F and W437X mutations which are located outside of the PINK1 kinase domain were recently shown to impair the ability of PINK1 to localize on the OMM of depolarized mitochondria [44], indicating that mutation-induced loss of mitochondrial damage-sensing function of PINK1 is another mechanism that triggers impaired mitochondrial quality control. In parkin, PD-linked L283P mutation found in the phospho-Ser65-ubiquitin-binding interface has been shown to impair parkin recruitment and activation on depolarized mitochondria [72–75], indicating that mutation-induced loss of phospho-Ser65-ubiquitin-binding ability of parkin can also lead to impaired mitochondrial quality control and neurodegeneration. Furthermore, a number of PD-linked mutations found in the parkin E3 ligase domain, such as parkin T240R and G430D mutations, are able to disrupt the E3 ligase activity of parkin for ubiquitinating its substrates and the ability of parkin to promote mitophagy [35, 50, 68], indicating that mutation-induced loss of parkin catalytic activity is another mechanism that causes impaired mitochondrial quality control and familial PD pathogenesis.

Human genetic studies have also identified a number of heterozygous mutations in PINK1 and parkin as risk factors for developing late-onset PD [9, 25–27]. In addition, oxidative damage to parkin has been detected in brains from patients with sporadic PD [61–63]. These findings suggest that dysregulation of PINK1/parkin-mediated, ubiquitin phosphorylation-dependent mitochondrial quality control may also contribute to the pathogenesis of sporadic PD.

### Ubiquitin phosphorylation as a potential biomarker for PD diagnosis

A major challenge in the PD field is to identify biomarkers for PD diagnosis, particularly at the early stage of the disease. The recent finding of a link between ubiquitin phosphorylation and PD pathogenesis suggests the possibility of using phospho-Ser65-ubiquitin as a potential PD biomarker. One approach for detecting phospho-Ser65-ubiquitin is to use anti-phospho-Ser65-ubiquitin antibodies that specifically recognize the Ser65-phosphorylated form of ubiquitin but not the non-phosphorylated form of ubiquitin. Such antibodies have recently been generated and used for analyses of human postmortem brain samples to show the accumulation of phospho-Ser65-ubiquitin in cytoplasmic granules that were localized adjacent to, but not within the Lewy bodies and Lewy neurites - the pathological hallmarks of PD [83]. The phospho-Ser65-ubiquitin-positive granules were partially co-localized with mitochondrial and lysosomal markers [83], suggesting that phospho-Ser65-ubiquitin was accumulated in damaged mitochondria and/or in autolysosomes containing damaged mitochondria, perhaps as a result of increased mitochondrial damage and/or impaired mitophagy. The phospho-Ser65-ubiquitin-positive granules appear to increase with aging and sporadic PD [83], providing support for the potential of phospho-Ser65-ubiquitin as a biomarker for PD.

In addition to the anti-phospho-Ser65-ubiquitin antibody approach, a sensitive, quantitative proteomic approach was recently developed to measure the levels of phospho-Ser65-ubiquitin in cell and tissue lysates [57, 79, 84]. This approach has been used to show increased brain levels of phospho-Ser65-ubiquitin in a mouse model of mitochondria dysfunction caused by enhanced mitochondrial DNA mutagenic stress [84], indicating that accumulation of phospho-Ser65-ubiquitin could occur as a result of increased mitochondrial damage *in vivo*. The sensitivity and quantitative nature of the proteomic approach are particularly useful for its further development as a diagnostic tool for PD.

### Targeting ubiquitin phosphorylation-dependent mitophagy for PD therapeutic development

Recent advances in understanding ubiquitin phosphorylation-dependent mitochondrial quality control have identified several potential targets for therapeutic intervention in PD. An attractive therapeutic target is the PINK1 kinase activity because PINK1-mediated phosphorylation of ubiquitin and other substrates is critically involved in neuroprotection against mitochondrial dysfunction [40, 54–56, 69, 85]. A novel PINK1-targeting approach was recently developed, which uses the ATP analogue kinetin triphosphate (KTP) or the KTP precursor kinetin to enhance the kinase activity of PINK1 [46]. Augmentation of PINK1 kinase activity by KTP or kinetin is able to promote parkin recruitment to damaged mitochondria and enhance cellular defense against oxidative stress-induced apoptosis [46], providing support for the therapeutic potential of PINK1 activation in PD treatment.

The E3 ubiquitin-protein ligase activity of parkin is another potential target for PD therapeutic development. The finding of phospho-Ser65-ubiquitin as an allosteric activator of parkin E3 ligase activity [72–75] that disrupts the autoinhibited conformation of parkin [64, 76] provides structural information for the development of small molecules that mimic the effect of phospho-Ser65-ubiquitin to activate parkin-dependent mitophagy. In addition, a recent study reported that parkin recruitment to damaged mitochondria is positively regulated by the deubiquitinating enzyme USP8 through its action to remove K6-linked ubiquitin chains from parkin [86], suggesting that USP8 activation could be another approach to promote clearance of damaged mitochondria. Furthermore, emerging data indicate that parkin-mediated mitochondrial protein ubiquitination and mitophagy are negatively regulated by deubiquitinating enzymes USP15 [87] and USP30 [88], which catalyze the removal of of ubiquitin from parkin substrates on damaged mitochondria. Knockdown of endogenous USP15 or USP30 is able to ameliorate mitochondrial and motor behavioral defects in parkin-deficient flies [87, 88] and enhance neuroprotection against paraquat toxicity [88], suggesting that USP15 and USP30 inhibition could provide potential therapeutic benefits for treating PD.

Phenotypic screening for chemical or genetic modifiers of PINK1 or parkin mutant phenotypes has emerged as a useful approach for PD drug discovery. Recent screens of a 2000-compound library using fibroblast cells from PD patients carrying parkin mutations have identified 15 compounds that can rescue mitochondrial dysfunction phenotypes of parkin-mutant patient cells [89]. Two of these compounds, ursocholanic acid and ursodeoxycholic acid, were further characterized and shown to ameliorate mitochondrial functional defects in parkin-mutant patient fibroblasts as well as in LRRK2-mutant patient fibroblasts, by acting through the glucocorticoid receptor and Akt signalling [89]. In addition, a *Drosophila* genetic screen using PINK1-deficient flies has identified UBIAD1/Heix, an enzyme involved in the synthesis of vitamin K_2_, as a modifier of PINK1 mutant phenotype [90]. Further analyses showed that vitamin K_2_ is able to improve the defective mitochondrial and behavioral phenotypes of PINK1 and parkin mutant flies, by acting as an electron carrier downstream of complex I [90]. These results support the therapeutic potential of vitamin K_2_ supplementation in PD treatment.

### Conclusions

An exciting, recent breakthrough from studying PD-linked proteins PINK1 and parkin is the discovery of a novel neuroprotective pathway in which PINK1 phosphorylates ubiquitin to activate parkin and promote mitophagy for maintaining mitochondrial and neuronal homeostasis. Emerging evidence has begun to indicate a link between the impairment of this neuroprotective pathway and the pathogenesis of familial PD as well as sporadic PD. These new mechanistic insights have revealed promising, novel avenues for PD diagnostic development and therapeutic intervention. Recent work supports the development of phospho-Ser65-ubiquitin as a potential biomarker for PD, and future studies using the quantitative proteomic approach [79, 84] to analyze phospho-Ser65-ubiquitin levels in PD patient samples, particularly in easily accessible body fluids such as blood or urine, should be pursued actively. In addition, newly gained insights into ubiquitin phosphorylation-dependent mitochondrial quality control have identified a number of potential therapeutic targets, which could be used in high-throughput screening for PD drug discovery. Furthermore, phenotypic screens for chemical or genetic modifiers of PINK1 and parkin mutant phenotypes have generated promising hits [89, 90], and future phenotypic screens using induced pluripotent stem cells (iPSCs) from PD patients will facilitate the development of new therapeutics to combat mitochondrial dysfunction and neurodegeneration in PD.
